# *Fusarium* Species Associated with Cherry Leaf Spot in China

**DOI:** 10.3390/plants11202760

**Published:** 2022-10-19

**Authors:** Yueyan Zhou, Wei Zhang, Xinghong Li, Shuxian Ji, Kandawatte Wedaralalage Thilini Chethana, Kevin David Hyde, Jiye Yan

**Affiliations:** 1 Beijing Key Laboratory of Environment Friendly Management on Fruit Diseases and Pests in North China, Institute of Plant Protection, Beijing Academy of Agriculture and Forestry Sciences, Beijing 100097, China; 2 Center of Excellence in Fungal Research, School of Science, Mae Fah Luang University, Chiang Rai 57100, Thailand

**Keywords:** *Prunus avium*, *Fusarium*, phylogeny, morphology, pathogenicity

## Abstract

Sweet cherry is an important fruit crop in China with a high economic value. From 2019 to 2020, a leaf spot disease was reported, with purplish-brown circular lesions in three cultivating regions in China. Twenty-four *Fusarium* isolates were obtained from diseased samples and were identified based on morphological characteristics and multi-locus phylogenetic analyses. Seven species, including *F. luffae* (7 isolates), *F. lateritium* (6 isolates), *F. compactum* (5 isolates), *F. nygamai* (2 isolates), *F. citri* (2 isolates), *F. ipomoeae* (1 isolate) and *F. curvatum* (1 isolate) were identified. The pathogenicity test showed that analyzed strains of all species could produce lesions on detached cherry leaves. Therefore, *Fusarium* was proved to be a pathogen of cherry leaf spots in China. This is the first report of *F. luffae*, *F. compactum*, *F. nygamai*, *F. citri*, *F. ipomoeae* and *F. curvatum* on sweet cherry in China.

## 1. Introduction

Sweet cherry (*Prunus avium* L.) is an economically important fruit crop widely planted in temperate regions worldwide. The cherry industry in China developed rapidly in the last decade, with 233,000 ha of cultivation area and a yield of 1,700,000 tons in 2019 [1,2]. Even with ideal conditions, various diseases occur on cherries, among which leaf spot is one of the most common and widespread [3]. Since the first report from the USA in 1878, the disease has spread quickly and occurred in most growing areas around the world [4]. The disease causes premature defoliation of leaves, the reduction of tree vigor and winter hardiness, and even tree death, leading to a bad quality of cherries [5]. *Blumeriella jaapii* was regarded as the causal agent of cherry leaf spot disease in Europe and North America [6]. In addition, several other associated fungi were reported. In Israel, the pathogen of cherry leaf spot was identified as *Cercospora circumscissa*, which caused a 40% yield loss in 1975 [7]. Additionally, *Alternaria alternata* and *Pseudocercospora pruni-persicicola* were reported as causing leaf spot in Greece and Korea, respectively [8,9]. In China, *Alternaria cerasi* and *Passalora circumscissa* were identified to cause “black spot” and “brown spot” of sweet cherry according to their morphology in the early days, respectively [10,11]. In recent years, more pathogenic species have been reported based on morphological characterization coupled with phylogenetic analysis, including four *Alternaria* species, three *Colletotrichum* species and four Didymellaceae species [3,12,13]. In this study, *Fusarium* spp. were isolated from cherry leaf spots for the first time, which supplemented the pathogen variety of the disease.

## 2. Results

### 2.1. Symptom Observation, Sample Collection and Fungal Isolation

**Figure 1:**
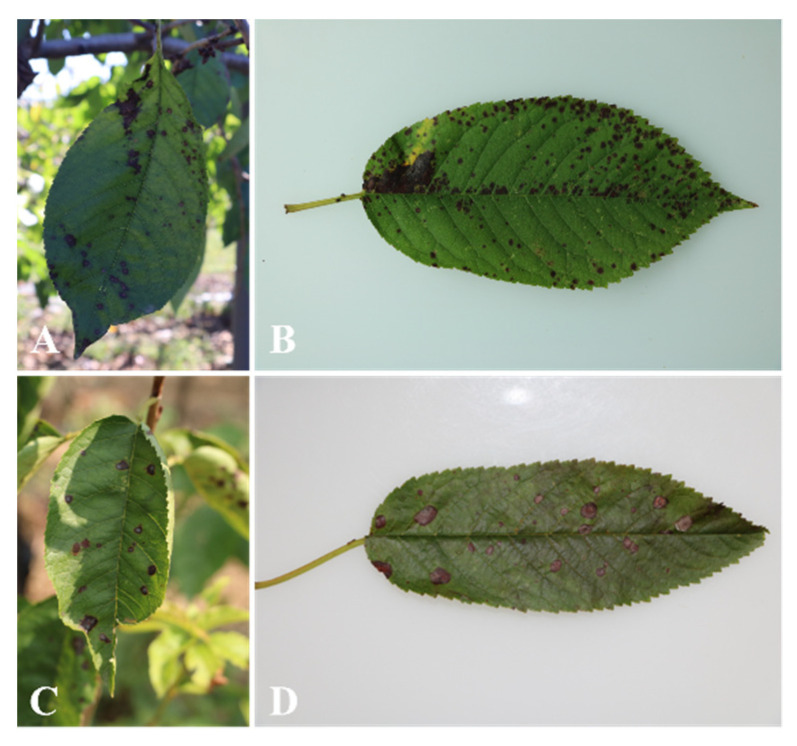
Symptoms of cherry leaf spot. (**A**,**B**) symptom type 1; (**C**,**D**) symptom type 2.

**Figure 2:**
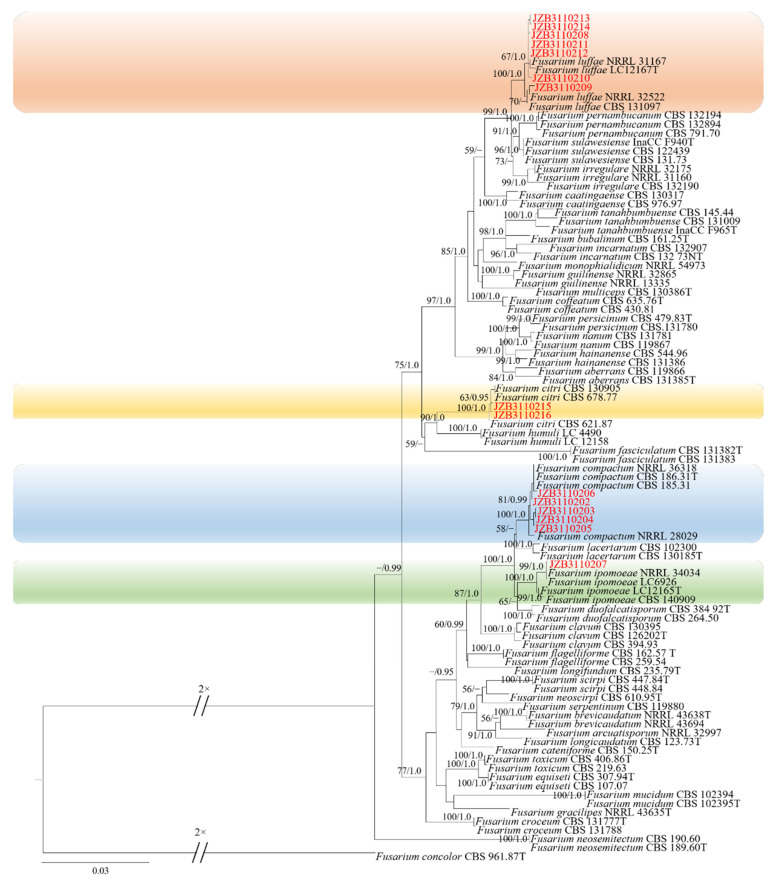
Phylogenetic tree generated by maximum likelihood analysis (RAxML) of FIESC based on the combined *CaM*, *rpb2* and *tef1* sequence data. The tree is rooted with *Fusarium concolor* (CBS 961.87). The best-scoring RAxML tree with a final likelihood value of –11,974.052080 is presented. The matrix had 701 distinct alignment patterns, with 7.69% of undetermined characters or gaps. Estimated base frequencies were as follows: A = 0.258901, C = 0.260331, G = 0.245699, T = 0.235069; substitution rates AC = 1.002698, AG = 4.065670, AT = 1.349366, CG = 0.882966, CT = 10.506153, GT = 1.000000; gamma distribution shape parameter α = 0.751525. ML bootstrap support values ≥50% and Bayesian posterior probabilities (BYPP) ≥ 0.95 are given near the nodes. The scale bar indicates 0.03 changes per site. Isolates belonging to this study are given in red.

Fifteen leaf spot samples were collected from sweet cherry trees cultured in open fields with the following symptoms, (1) small purple-brownish spots which may merge with expansion (Figure 1A,B); (2) circular or irregular brownish gray necrotic lesions with dark brown margin (Figure 1C,D). Twenty-four *Fusarium* isolates were obtained by tissue isolation, among which 13 were from Beijing, 9 from Shandong and 2 from Liaoning (Table 1).

### 2.2. Molecular Characterization and Phylogenetic Analysis

The ITS sequences of isolates showed 100% similarity to *Fusarium* spp. based on BLAST analysis. Reference sequences of *Fusarium* were downloaded from the NCBI database (Table S1). Phylogenic trees of *Fusarium incarnatum-equiseti* species complex (FIESC), *Fusarium lateritium* species complex (FLSC), *Fusarium fujikuroi* species complex (FFSC) and *Fusarium oxysporum* species complex (FOSC) were constructed using combined sequence alignment. The sequences were deposited in the GenBank, and accession numbers were obtained (Table 1).

Phylogenetic analysis of FIESC was constructed based on the combined *CaM*, *rpb2* and *tef1* multi-locus dataset, with *Fusarium concolor* (CBS 961.87) as the outgroup taxon (Figure 2). Both maximum likelihood and Bayesian analyses resulted in the same topology. Among the ninety-six fungal isolates in the dataset, fifteen were from the current study. Seven isolates clustered with *F. luffae* in two branches with 67% ML bootstrap and 1.0 BYPP, and 70% ML bootstrap, respectively, five clustered with *F. compactum* with 81% ML bootstrap and 0.99 BYPP, two clustered with *F. citri* with 63% ML bootstrap and 0.95 BYPP, and one isolate clustered with *F. ipomoeae* with 99% ML bootstrap and 1.0 BYPP.

**Figure 3:**
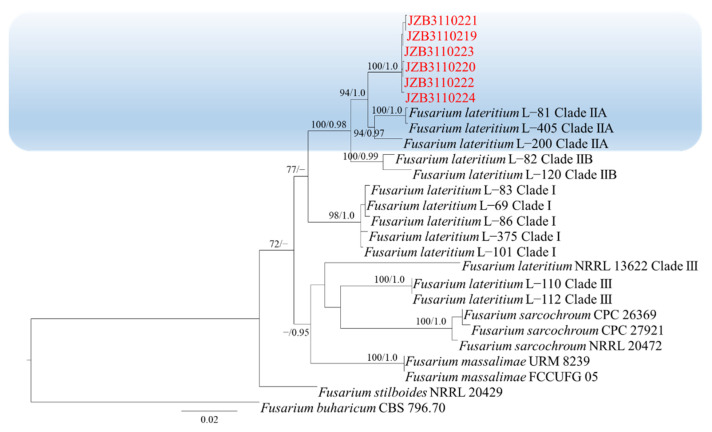
Phylogenetic tree generated by maximum likelihood analysis (RAxML) of FLSC based on the combined *rpb2*, *tef1* and *tub2* sequence data. The tree is rooted with *Fusarium buharicum* (CBS 796.70). The best-scoring RAxML tree with a final likelihood value of −5602.556442 is presented. The matrix had 358 distinct alignment patterns, with 32.15% being undetermined characters or gaps. Estimated base frequencies were as follows: A = 0.236754, C = 0.282131, G = 0.242563, T = 0.238552; substitution rates AC = 2.260928, AG = 7.239234, AT = 2.341489, CG = 1.331525, CT = 16.787035, GT = 1.000000; gamma distribution shape parameter α = 1.252177. ML bootstrap support values ≥50% and Bayesian posterior probabilities (BYPP) ≥ 0.95 are given near the nodes. The scale bar indicates 0.02 changes per site. Isolates belonging to this study are given in red.

Phylogenetic analysis of FLSC was constructed based on combined *rpb2*, *tef1* and *tub2* multi-locus dataset, with *Fusarium buharicum* (CBS 796.70) as the outgroup taxon (Figure 3). Both maximum likelihood and Bayesian analyses resulted in the same topology. Six isolates clustered with *F. lateritium* with 94% ML bootstrap and 1.0 BYPP.

**Figure 4:**
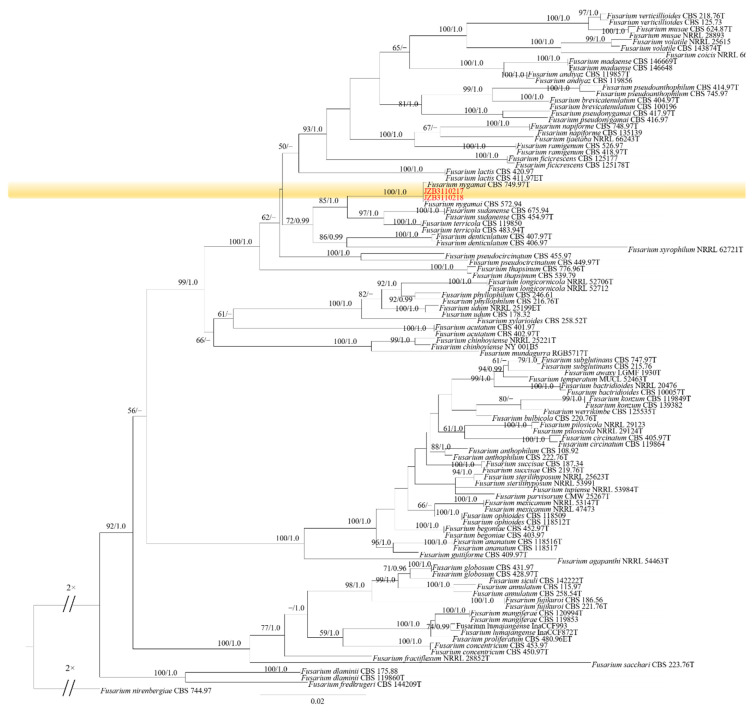
Phylogenetic tree generated by maximum likelihood analysis (RAxML) of FFSC based on the combined *tef1*, *rpb2*, *tub2* and *CaM* sequence data. The tree is rooted with *Fusarium nirenbergiae* (CBS 744.97). The best-scoring RAxML tree with a final likelihood value of –16,169.804811 is presented. The matrix had 1172 distinct alignment patterns, with 12.61% of undetermined characters or gaps. Estimated base frequencies were as follows: A = 0.246851, C = 0.265429, G = 0.243377, T = 0.244344; substitution rates AC = 1.195289, AG = 4.703565, AT = 1.258970, CG = 0.717390, CT = 9.004544, GT = 1.000000; gamma distribution shape parameter α = 0.838651. ML bootstrap support values ≥50% and Bayesian posterior probabilities (BYPP) ≥ 0.95 are given near the nodes. The scale bar indicates 0.03 changes per site. Isolates belonging to this study are given in red.

Phylogenetic analysis of FFSC was constructed based on the combined *tef1*, *rpb2*, *tub2* and *CaM* multi-locus dataset, with *Fusarium nirenbergiae* (CBS 744.97) as the outgroup taxon (Figure 4). Both maximum likelihood and Bayesian analyses resulted in the same topology. Two isolates clustered with *F. nygamai* with 100% ML bootstrap and 1.0 BYPP. 

**Figure 5:**
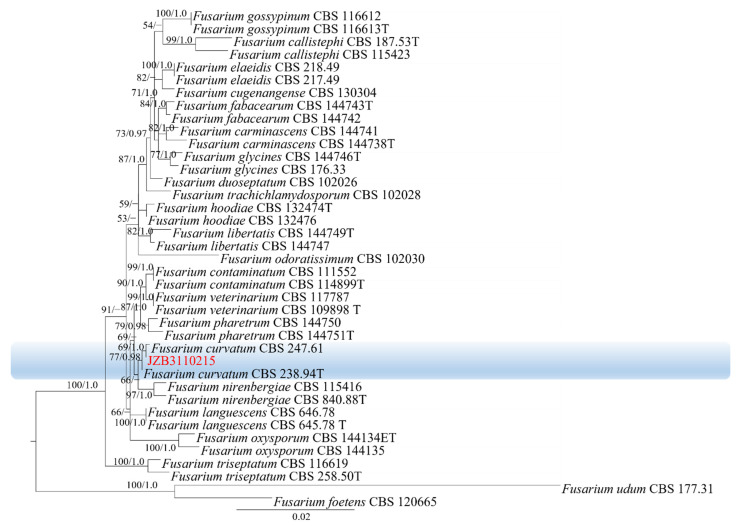
Phylogenetic tree generated by maximum likelihood analysis (RAxML) of FOSC based on the combined *rpb2*, *tef1* and *tub2* sequence data. The tree is rooted with *Fusarium foetens* (CBS 120665) and *Fusarium udum* (CBS 177.31). The best-scoring RAxML tree with a final likelihood value of −4987.801452 is presented. The matrix had 199 distinct alignment patterns, with 0.90% of undetermined characters or gaps. Estimated base frequencies were as follows: A = 0.251044, C = 0.269288, G = 0.238899, T = 0.240769; substitution rates AC = 1.155437, AG = 2.887244, AT = 0.424595, CG = 0.728892, CT = 5.651969, GT = 1.000000; gamma distribution shape parameter α = 1.022063. ML bootstrap support values ≥50% and Bayesian posterior probabilities (BYPP) ≥ 0.95 are given near the nodes. The scale bar indicates 0.02 changes per site. Isolates belonging to this study are given in red.

Phylogenetic analysis of FOSC was constructed based on the combined *rpb2*, *tef1* and *tub2* multi-locus dataset, with *Fusarium foetens* (CBS 120665) and *Fusarium udum* (CBS 177.31) as the outgroup taxa (Figure 5). Both maximum likelihood and Bayesian analyses resulted in the same topology. One isolate clustered with *F. curvatum* with 69% ML bootstrap and 1.0 BYPP.

### 2.3. Morphological Observation

**Figure 6:**
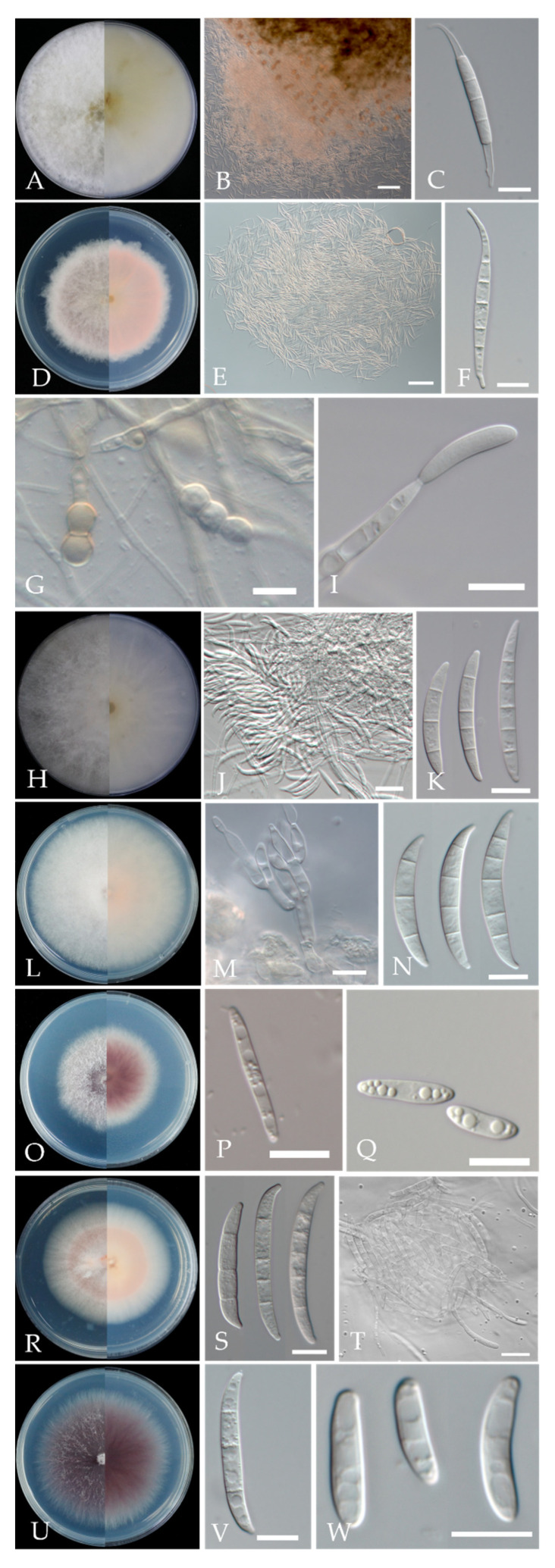
Morphological characteristics of *Fusarium* species isolated from cherry leaf spot. (**A**) Colony of *F. compactum*; (**B**) Sporodochia of *F. compactum* on carnation leaf; (**C**) Macroconidia of *F. compactum* on CLA; (**D**) Colony of *F. ipomoeae*; (**E**) Sporodochia of *F. ipomoeae*; (**F**) Macroconidia of *F. ipomoeae*; (**G**) Chlamydospores of *F. ipomoeae*; (**H**) Colony of *F. luffae*; (**I**) Conidiophore of *F. luffae* on aerial hyphae; (**J**) Sporodochia of *F. luffae*; (**K**) Macroconidia of *F. luffae*; (**L**) Colony of *F. citri*; (**M**) Conidiogenous cells of *F. citri* formed on sporodochia; (**N**) Macroconidia of *F. citri*; (**O**) Colony of *F. nygamai*; (**P**) Macroconidia of *F. nygamai*; (**Q**) Microconidia of *F. nygamai*; (**R**) Colony of *F. lateritium*; (**S**) Macroconidia of *F. lateritium*; (**T**) Sporodochia of *F. lateritium*; (**U**) Colony of *F. curvatum*; (**V**) Macroconidia of *F. curvatum*; (**W**) Microconidia of *F. curvatum*. scale bar: (**A**,**C**,**D**,**F**–**I**,**K**–**S**,**U**–**W**) = 10 μm; (**J**,**T**) = 20 μm; (**B**,**E**) = 100 μm.

Morphological characteristics were shown in Figure 6, Table 2 and Table 3. Based on cultural and morphological characters, *Fusarium* isolates were consistent with *F. compactum*, *F. ipomoeae*, *F. luffae*, *F. citri*, *F. nygamai*, *F. lateritium* and *F. curvatum,* as previously described [14,15,16].

### 2.4. Pathogenicity Test

**Figure 7:**
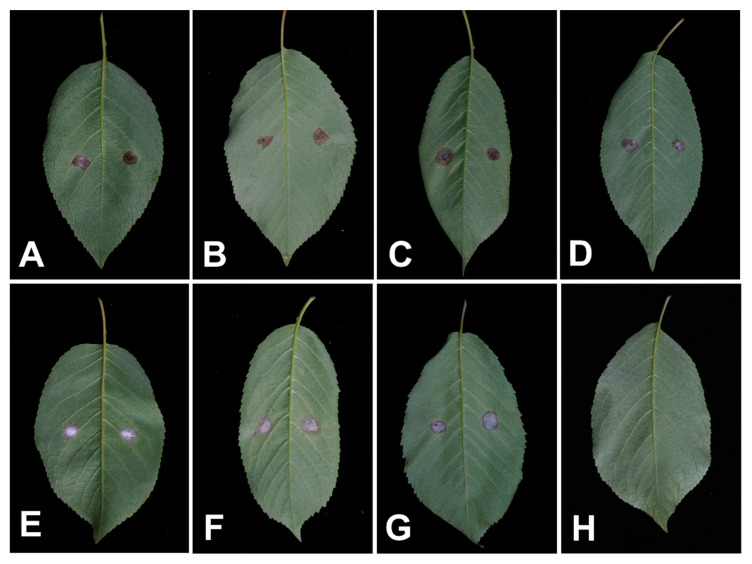
The pathogenicity test of *Fusarium* spp. on detached cherry leaves three days post-inoculation. (**A**–**G**) Detached leaves inoculation of *F. luffae*, *F. lateritium*, *F. compactum*, *F. nygamai*, *F. citri*, *F. ipomoeae and F. curvatum*, respectively. Left side of the leaf was wounded before inoculation, right side was unwounded; (**H**) Control.

Three days post-inoculation, all *Fusarium* isolates were pathogenic to detached cherry leaves, resulting in lesions similar to the disease symptoms observed in the field (Figure 7). The disease incidences [(symptomatic sites/total inoculated sites) × 100%] and lesion diameter of wounded and unwounded leaves of each species are reported in Table 4. No symptoms were observed on the control leaves. According to the result, *F. compactum*, *F. luffae* and *F. ipomoeae* were the most aggressive and caused lesion diameters of more than 10 mm. The other species were relatively less virulent, with lesion diameters varying from 7 mm to 10 mm, and lesion diameters did not differ significantly. The disease incidence of wounded leaves was slightly higher than that of unwounded leaves, while unwounded leaves of some species appeared to form larger lesion diameters. The fungi were re-isolated from the lesions successfully and identified using morphology and molecular analyses. 

## 3. Discussion

Leaf spot is a common and severe disease of sweet cherry caused by various pathogens, while the occurrence of *Fusarium* on sweet cherry has not been reported before in the world. *Fusarium* is one of the most renowned genera, which includes a large number of saprotrophs, endophytes or pathogens [17]. As a common plant pathogen, *Fusarium* spp. can cause many diseases with a wide range of hosts, usually causing wilts, blights, rots, and cankers (Ma et al., 2013). *Fusarium* can also be the causal agent of leaf spot disease in several plants including Dracaena, mango, peanut and *Bletilla striata* [18,19,20,21]. 

*Fusarium* species reported on sweet cherry are usually associated with trunk disease. *Fusarium oxysporum* has been reported as the pathogen of cherry root and crown rot in Canada [22], and *F. lateritium* was reported to cause trunk diseases in Australia [23]. Five *Fusarium* species have also been reported to cause postharvest rot on Chinese cherry [24]. In this study, *Fusarium* species were identified as causal agents of cherry leaf spot based on morphological characteristics and molecular phylogenetic analyses. Among them, seven were pathogenic to cherry leaves; specifically, *F. compactum*, *F. luffae* and *F. ipomoeae* showed relatively high virulence. 

*Fusarium compactum* had been regarded as a saprophyte, while being reported as the pathogen of corm and root rot of banana, a fatal canker on Italian cypress, and a wilt and root rot of peanuts [25,26,27]. *Fusarium ipomoeae*, *F*. *luffae* and *F*. *citri* were introduced as new species in the study reported on *Fusarium incarnatum-equiseti* complex from China [15]. Afterwards, *F*. *ipomoeae* was reported to cause leaf spots on peanut and *Bletilla striata* [20,21], *F*. *luffae* was reported to cause fruit rot on muskmelon in China [28], and *F. citri* was initially isolated from *Citrus* [15]. *Fusarium nygamai* was first observed on a basal stalk and root rot of grain sorghum [29] and can also cause root rot on other crops including cotton, maize, millet, rice and sorghum [14]. *Fusarium lateritium* can cause wilt, tip or branch dieback, or cankers on many woody trees and shrubs including some important fruit trees and coffee [14]. These species all have records as pathogens; therefore, attention should be paid to the Fusarium leaf spot on sweet cherry plant, which may cause severe damage in some conditions.

Relevant results in the current study expand the pathogenic fungal species on sweet cherry, and further research is necessary to understand the influence of *Fusarium* on cherry diseases.

## 4. Materials and Methods

### 4.1. Sample Collection and Isolation of Fungal Strains

From 2019 to 2020, cherry leaf spot samples were collected from farmers’ fields in Beijing, Liaoning and Shandong Provinces in China. Each sample contains three leaves from a tree. Lesions were selected randomly and diseased tissues at the junction of disease and healthy region were cut into 5 × 5 mm squares, then surface-sterilized using 75% ethanol for 30 s and washed three times using sterile water. After drying on sterilized filter paper, tissues were transferred to potato dextrose agar (PDA) plates and incubated at 25 °C, and the hyphae from colony margins were transferred to fresh PDA plates after five days. After sporulation, single-spore isolation was conducted to get purified cultures.

### 4.2. DNA Extraction, PCR Amplification and Phylogenetic Analysis

Mycelia were collected from the cultures on PDA media for seven days, and genomic DNA was extracted using the CTAB method. The internal transcribed spacer (ITS) region was amplified first using the primers ITS5/ITS4 [30], and sequences were searched using BLASTn (Basic Local Alignment Search Tool) on NCBI (National Center for Biotechnology Information) to identify reference sequences. Then, the RNA polymerase second largest subunit (*rpb2*), translation elongation factor 1-alpha (*tef-1*), beta-tubulin (*tub2*) and calmodulin (*CaM*) genes were amplified, and the primers were listed in Table 5. The 50 μL reaction system for amplification contained 25 μL 2 × Taq PCR Master Mix (Biomed, Beijing, China), 21 μL double-distilled water, 1 μL of each forward and reverse primers (10 μM) and 2 μL DNA template. PCR conditions were as follows: initial denaturation at 94 °C for 3 min, followed by 34 cycles of denaturation at 94 °C for 30 s, primer annealing at the temperatures for 1 min and extension at 72 °C for 1 min, and final extension at 72 °C for 10 min. The PCR products were examined in 1.5% agarose gel and sequenced by Tsingke Biotech Co., Ltd. (Beijing, China). The sequences generated in the current study and reference sequences of *Fusarium* species downloaded from the GenBank database of NCBI (Table S1) were aligned with MAFFT v. 7 (https://mafft.cbrc.jp/alignment/server/ (accessed on 18 March 2022) [31]. Aligned gene regions were adjusted manually where necessary and combined with BioEdit 7.0.9.0.

Maximum likelihood (ML) phylogenic analyses of species complexes were conducted using RAxML-HPC2 on XSEDE (v8.2.8) through the CIPRES Science Gateway (https://www.phylo.org/portal2 (accessed on 13 April 2022) [32]. The evolutionary model GTR + I + G was applied to all gene regions, and bootstrap support values were obtained by running 1000 pseudo replicates.

The Bayesian Inference (BI) analyses were performed based on the Markov Chain Monte Carlo sampling (BMCMC) method using MrBayes v3.1.2 to evaluate posterior probabilities (BYPP) [33]. Six simultaneous Markov chains were run for 2,000,000 generations and trees were sampled at every 1000th generation.

Phylogenetic trees were visualized with FigTree v1.4.0 and edited in Microsoft Office PowerPoint 2016. The ML bootstrap supports greater than or equal to 50% and Bayesian posterior probabilities greater than or equal to 0.95 are shown at the nodes in the resulting phylogenetic tree. The sequences generated in this study were deposited in the GenBank.

### 4.3. Morphological Identification

The isolates were cultured on PDA and carnation leaf agar (CLA) to observe colony and conidia morphology respectively [14]. Mycelial discs of 4 mm diam were inoculated at the center of PDA plates and incubated in the dark at 25 °C. Colony diameters were measured after five days, and cultural features were examined and photographed. Isolates inoculated on CLA were cultured under 12/12 h light/dark cycle conditions for 10 days, the characteristics of micro- and macroconidia were observed under Axiocam 506 color Imager Z2 photographic microscope (Carl Zeiss Microscopy GmbH, Jena, Germany) and measurements were made with ZEN Pro 2012 (Carl Zeiss Microscopy).

### 4.4. Pathogenicity Assays

A pathogenicity test was conducted by inoculating detached leaves with spore suspension. Representative isolates of each species were cultured on PDA at 25 °C until sporulation. Tender, healthy-looking leaves of *P. avium* cv. ‘Tieton’ were collected from Tongzhou Experimental Station for Cherries, Beijing Academy of Forestry and Pomology Sciences, Beijing, China. Leaves were surface sterilized with 75% ethanol for 30 s, rinsed three times with sterile water and air-dried on sterilized filter paper. Spores collected from cultures were dispersed in sterile water and the concentration was adjusted to 1.0 × 10^6^/mL using a hemocytometer. Four small holes were pierced on the left side of the vein by a sterile needle for the wounded inoculation. Then, 20 μL spore suspension was inoculated on the wounds and non-wound treatment was performed on symmetrical halves of each leaf. Six leaves were inoculated for each isolate with three replicates. Sterile water was used as the control. Leaves were incubated in disinfected plastic boxes at 25 °C and 80% relative humidity, with a 12/12 h light/dark cycle. Disease incidence (%) [(symptomatic sites/total inoculated sites) × 100%] was calculated and lesion diameters were measured after the appearance of symptoms. The fungal strains used for leaves inoculation were re-isolated to confirm Koch’s postulates. Data were subjected to a one-way analysis of variance (ANOVA) using the software IBM SPSS Statistics v20 to determine the significance of the differences. Means of different species were separated using the least significant difference test at the *p* = 0.05 level. 

## Figures and Tables

**Table 1:** Information and GenBank accessions of *Fusarium* isolates obtained in this study.

Species	Isolate Number	Origin	Date Collected	GenBank Accession			
				*CaM*	*rpb2*	*tef1*	*tub2*
*F. compactum*	JZB3110202	Beijing	2019.7	OP018566	OP018571	OP018576	
*F. compactum*	JZB3110203	Beijing	2019.8	OP018567	OP018572	OP018577	
*F. compactum*	JZB3110204	Beijing	2019.8	OP018568	OP018573	OP018578	
*F. compactum*	JZB3110205	Beijing	2019.8	OP018569	OP018574	OP018579	
*F. compactum*	JZB3110206	Liaoning	2019.9	OP018570	OP018575	OP018580	
*F. ipomoeae*	JZB3110207	Beijing	2019.8	OP018581	OP018582	OP018583	
*F. luffae*	JZB3110208	Beijing	2019.7	OP018545	OP018552	OP018559	
*F. luffae*	JZB3110209	Shandong	2019.8	OP018546	OP018553	OP018560	
*F. luffae*	JZB3110210	Shandong	2019.8	OP018547	OP018554	OP018561	
*F. luffae*	JZB3110211	Shandong	2019.8	OP018548	OP018555	OP018562	
*F. luffae*	JZB3110212	Shandong	2019.8	OP018549	OP018556	OP018563	
*F. luffae*	JZB3110213	Beijing	2019.10	OP018550	OP018557	OP018564	
*F. luffae*	JZB3110214	Beijing	2020.9	OP018551	OP018558	OP018565	
*F. citri*	JZB3110215	Shandong	2019.8	OP039326	OP039328		
*F. citri*	JZB3110216	Beijing	2019.10	OP039327	OP039329		
*F. nygamai*	JZB3110217	Beijing	2019.8	OP039354	OP039350	OP039348	OP039352
*F. nygamai*	JZB3110218	Beijing	2019.8	OP039355	OP039351	OP039349	OP039353
*F. lateritium*	JZB3110219	Shandong	2019.8		OP039330	OP039336	OP039342
*F. lateritium*	JZB3110220	Shandong	2019.8		OP039331	OP039337	OP039343
*F. lateritium*	JZB3110221	Shandong	2019.8		OP039332	OP039338	OP039344
*F. lateritium*	JZB3110222	Shandong	2019.8		OP039333	OP039339	OP039345
*F. lateritium*	JZB3110223	Shandong	2020.9		OP039334	OP039340	OP039346
*F. lateritium*	JZB3110224	Shandong	2020.9		OP039335	OP039341	OP039347
*F. curvatum*	JZB3110225	Liaoning	2019.9		OP039359	OP039360	OP039361

JZB: Culture collection of Institute of Plant Protection, Beijing Academy of Agriculture and Forestry Sciences, Beijing 100097, China.

**Table 2:** Characteristics of colonies of *Fusarium* fungi cultivated on PDA.

Species	Average Diameter of Colony (Range) on 5 d, mm	Type and Color of Aerial Mycelium	Reverse Pigmentation
*F. compactum*	79–85	White mycelia with brown pigments produced in the agar	White to pale yellow
*F. ipomoeae*	59–65	Colony margin lobate, pinkish white	Pale pink
*F. luffae*	67–73	Aerial mycelia dense, colony white	White to pale yellow
*F. citri*	78–80	Aerial mycelia dense, colony margin entire, white	Pinkish white
*F. nygamai*	53–56	Mycelia violet, with violet pigments produced in the agar	Violet with white margin
*F. lateritium*	45–54	Mycelia sparse, pale orange or pale pink	Pale orange or pale pink with white margin
*F. curvatum*	68–70	Mycelia violet, with violet pigments produced in the agar	Dark violet

**Table 3:** Description of conidia of *Fusarium* fungi cultivated on CLA.

Species	Macroconidia		Microconidia	
	Shape, Number of Septa	Average Size (Range)	Shape, Number of Septa	Average Size (Range)
*F. compactum*	Strong dorsiventral curvature, apical cell elongate and tapering that is often needle like in appearance, basal cell foot-shaped, usually 5-septate	43.04–62.05 × 4.02–5.13 μm (avg. = 54.08 × 4.50 μm, *n* = 50)	Not observed	
*F. ipomoeae*	Dorsiventral curvature, smooth, hyaline, apical cell hooked to tapering, basal cell foot-shaped. Usually 5-septate	40.72–66.64 × 3.67–4.65 μm (avg. = 56.60 × 4.16 μm, *n* = 50)	Not observed	
*F. luffae*	Falcate, slender, slightly curved, smooth to slightly rough, hyaline, apical cell blunt or hooked, basal cell barely notched, 3–5-septate	26.18–42.88 × 3.75–4.69 μm (avg. = 33.69 × 4.17 μm, *n* = 50)	Not observed	
*F. citri*	Falcate, hyaline, apical cell papillate to hooked, basal cell distinctly notched to foot-shaped, 3–5-septate	24.23–48.42 × 4.05–5.39 μm (avg. = 36.54 × 4.86 μm, *n* = 50)	Not observed	
*F. nygamai*	Slender, thin walled, hyaline, straight to slightly curved, apical cell short and tapered, basal cell notched or foot-shaped, usually 3-septate	21.08–36.30 × 2.36–3.91 μm (avg. = 25.81 × 2.36 μm, *n* = 30)	Oval to elliptical, usually 0-septate	10.06–16.18 × 2.23–3.86 μm (avg. = 12.88 × 3.02 μm, *n* = 30)
*F. lateritium*	Falcate to relatively straight, with parallel walls, apical cell hooked or beaked, basal cell foot-shaped or notched, 3–5-septate, usually 5-septate	38.81–59.69 × 4.31–5.60 μm (avg. = 49.10 × 4.88 μm, *n* = 50)	Not observed	

**Table 4:** Features of colonies and macroconidia for *Fusarium* species isolated in this study. (lowercase letters indicate the significant differences for lesion diameters (*p* < 0.05).

Species	Disease Incidence (%)		Lesion Diameter (mm)	
	Wounded	Unwounded	Wounded	Unwounded
*F. compactum*	95	95	13.5 ± 0.3 a	14.0 ± 0.3 a
*F. ipomoeae*	95	75	11.8 ± 0.3 ab	12.8 ± 0.3 a
*F. luffae*	100	95	12.8 ± 0.4 ab	14.5 ± 0.5 a
*F. citri*	100	75	8.3 ± 0.1 b	8.8 ± 0.1 b
*F. nygamai*	100	95	8.3 ± 0.1 b	7.5 ± 0.1 b
*F. lateritium*	80	90	9.3 ± 0.1 ab	7.0 ± 0.2 b
*F. curvatum*	100	100	10.0 ± 0.1 ab	7.5 ± 0.1 b

**Table 5:** Primers used in this study.

Gene/Region	Primer	Sequence (5′-3′)	Annealing Temperature	Reference
ITS	ITS5	GGAAGTAAAAGTCGTAACAAGG	58 °C	[30]
	ITS4	TCCTCCGCTTATTGATATGC		
*rpb2*	5F2	GGGGWGAYCAGAAGAAGGC	56 °C	[34]
	7Cr	CCCATRGCTTGYTTRCCCAT		[35]
*tef1*	EF1	ATGGGTAAGGARGACAAGAC	55 °C	[36]
	EF2	GGARGTACCAGTSATCATG		
*CaM*	CL1	GARTWCAAGGAGGCCTTCTC	55 °C	[37]
	CL2A	TTTTTGCATCATGAGTTGGAC		
*tub2*	T1	AACATGCGTGAGATTGTAAGT	52 °C	[38]
	T2	TAGTGACCCTTGGCCCAGTTG		

## Data Availability

All the newly produced sequences are deposited in the GenBank and the accession numbers are given in Table 1.

## Supplementary Materials

The following supporting information can be downloaded at: https://www.mdpi.com/article/10.3390/plants11202760/s1, Table S1: Sequences of used in the phylogenetic analyses with GenBank accession numbers.

## References

[1] Huang Z.G., Liu C.L., Li M., Zhao G.R., Li Y.H. (2014). The development situation of sweet cherry industry in China and abroad during recent two decades and prognostication for the future. J. Fruit Sci..

[2] Duan X.W., Li M., Tan Y., Zhang X.M., Wang B.G., Yan G.H., Wang J., Pan F.R., Liu Q.Z., Zhang K.C. (2019). Fruit scientific research in new China in the past 70 years: Cherry. J. Fruit Sci..

[3] Chethana K.W.T., Jayawardene R.S., Zhang W., Zhou Y.Y., Liu M., Hyde K.D., Li X.H., Wang J., Zhang K.C., Yan J.Y. (2019). Molecular characterization and pathogenicity of fungal taxa associated with cherry leaf spot disease. Mycosphere.

[4] Holb I.J. (2009). Some biological features of cherry leaf spot (*Blumeriella jaapii*) with special reference to cultivar susceptibility. Int. J. Hortic. Sci..

[5] Wharton P.S., Iezzoni A., Jones A.L. (2003). Screening cherry germ plasm for resistance to leaf spot. Plant Dis..

[6] Schuster M., Tobutt K.R. (2004). Screening of cherries for resistance to leaf spot, *Blumeriella jaapii*. Acta Hortic..

[7] Sztejnberg A. (1986). Etiology and control of cherry leaf spot disease in Israel caused by *Cercospora circumscissa*. Plant Dis..

[8] Thomidis T., Tsipouridis C. (2006). First report of Alternaria leaf spot on cherry trees in Greece. Plant Dis..

[9] Choi I.Y., Braun U., Park J.H., Shin H.D. (2014). First report of leaf spot caused by *Pseudocercospora Pruni-Persicicola* on sweet cherry in Korea. Plant Dis..

[10] Zhu J.L., Chang Y.Y. (2004). Identification and biological characterization of the pathogen causing cherry black target spot. China Fruits.

[11] Liu B.Y., Zhang W., Luan B.H., Wang Y.Z. (2012). Identification of pathogen and epidemic dynamics of brown spot of sweet cherry. J. Fruit Sci..

[12] Yang L.P., Jin M.J., Cui L.X., Li T.H., An J., Wei L.J., Chang T., Yang C.D. (2020). Isolation and identification of the pathogen causing cherry black spot in Gansu Province. J. Fruit Sci..

[13] Tang Z., Lou J., He L., Wang Q., Chen L., Zhong X., Wu C., Zhang L., Wang Z.Q. (2022). First Report of *Colletotrichum fructicola* causing anthracnose on cherry (*Prunus avium*) in China. Plant Dis..

[14] Leslie J.F., Summerell B.A. (2006). The Fusarium Laboratory Manual.

[15] Wang M.M., Chen Q., Diao Y.Z., Duan W.J., Cai L. (2019). *Fusarium incarnatum-equiseti* complex from China. Persoonia.

[16] Lombard L., Sandoval-Denis M., Lamprecht S.C., Crous P.W. (2019). Epitypification of *Fusarium oxysporum*—Clearing the taxonomic chaos. Persoonia.

[17] Sandoval-Denis M., Guarnaccia V., Polizzi G., Crous P.W. (2018). Symptomatic citrus trees reveal a new pathogenic lineage in *Fusarium* and two new *Neocosmospora* Species. Persoonia.

[18] Baka Z.A.M., Krzywinski K. (1996). Fungi associated with leaf spots of *Dracaena Ombet* (Kotschy and Peyr). Microbiol. Res..

[19] Guo Z., Yu Z., Li Q., Tang L., Guo T., Huang S., Mo J., Hsiang T., Luo S. (2021). *Fusarium* species associated with leaf spots of mango in China. Microb. Pathogenesis.

[20] Xu M., Zhang X., Yu J., Guo Z., Li Y., Wu J., Chi Y. (2021). First report of *Fusarium ipomoeae* causing peanut leaf spot in China. Plant Dis..

[21] Zhou L.Y., Yang S.F., Wang S.M., Lv J.W., Wan W.Q., Li Y.H., Zhou H. (2021). Identification of *Fusarium ipomoeae* as the causative agent of leaf spot disease in *Bletilla Striata* in China. Plant Dis..

[22] Úrbez-Torres J.R., Boulé J., Haag P., Hampson C., O’Gorman D.T. (2016). First report of root and crown rot caused by *Fusarium oxysporum* on sweet cherry (*Prunus avium*) in British Columbia. Plant Dis..

[23] Cook R.P., Dubé A.J. (1989). Host-Pathogen Index of Plant Diseases in South Australia.

[24] Wang C., Wang Y., Wang L., Li X., Wang M., Wang J. (2021). *Fusarium* species causing postharvest rot on Chinese cherry in China. Crop Prot..

[25] Frisullo S.A., Logrieco A., Moretti A., Grammatikaki G., Bottalico A. (1994). Banana corm and root rot by *Fusarium*
*compactum* in Crete. Phytopatho. Mediterr..

[26] Madar Z., Kimchi M., Solel Z. (1996). Fusarium canker of Italian cypress. Eur. J. Forest Pathol..

[27] Saleh O.I. (1997). Wilt, root rot and seed diseases of groundnut in El-Minia governorate, Egypt. Egypt. J. Phytopathol..

[28] Zhang X.P., Cao X.D., Dang Q.Q., Liu Y.G., Zhu X.P., Xia J.W. (2022). First report of fruit rot caused by *Fusarium luffae* in muskmelon in China. Plant Dis..

[29] Trimboli D.S., Burgess L.W. (1985). Fungi associated with basal stalk rot and root rot of dryland grain sorghum in New South Wales. Plant Prot. Q..

[30] White T.J. (1990). Amplification and direct sequencing of fungal ribosomal RNA genes for phylogenetics. PCR Protoc. Guide Methods Appl..

[31] Katoh K., Rozewicki J., Yamada K.D. (2019). MAFFT online service: Multiple sequence alignment, interactive sequence choice and visualization. Brief. Bioinform..

[32] Miller M.A., Pfeiffer W., Schwartz T. Creating the CIPRES Science Gateway for inference of large phylogenetic trees. Proceedings of the 2010 Gateway Computing Environments Workshop (GCE).

[33] Ronquist F., Huelsenbeck J.P. (2003). MrBayes 3: Bayesian phylogenetic inference under mixed models. Bioinformatics.

[34] Reeb V., Lutzoni F., Roux C. (2004). Contribution of *RPB2* to multilocus phylogenetic studies of the euascomycetes (Pezizomycotina, Fungi) with special emphasis on the lichen-forming Acarosporaceae and evolution of polyspory. Mol. Phylogenet. Evol..

[35] Liu Y.J., Whelen S., Hall B.D. (1999). Phylogenetic relationships among ascomycetes: Evidence from an RNA polymerase II subunit. Mol. Biol. Evol..

[36] O’Donnell K., Kistler H.C., Cigelnik E., Ploetz R.C. (1998). Multiple evolutionary origins of the fungus causing panama disease of banana: Concordant evidence from nuclear and mitochondrial gene genealogies. Proc. Natl. Acad. Sci. USA.

[37] O’Donnell K., Kistler H.C., Tacke B.K., Casper H.H. (2000). Gene genealogies reveal global phylogeographic structure and reproductive isolation among lineages of *Fusarium graminearum*, the fungus causing wheat scab. Proc. Natl. Acad. Sci. USA.

[38] O’Donnell K., Cigelnik E. (1997). Two divergent intragenomic rDNA ITS2 types within a monophyletic lineage of the fungus *Fusarium* are nonorthologous. Mol. Phylogenet. Evol..

