# Supplementary material for: Fusarium Species Associated with Cherry Leaf Spot in China
Source: Plants (Basel). 2022 Oct 19;11(20):2760. doi: 10.3390/plants11202760 (PMC9609575; doi:10.3390/plants11202760)
Supplement: Supplementary file 1 [file plants-11-02760-s001.zip › plants-1869382-supplementary.pdf]

Table S1. Sequences of used in the phylogenetic analyses with GenBank accession numbers

Species	Culture accession	GenBank accession			
		CaM	*rpb2*	*tef1*	*tub2*
F. aberrans	CBS 119866	MN170310	MN170377	MN170444	
F. aberrans	CBS131385T	MN170311	MN170378	MN170445	
F. acutatum	CBS 401.97	MW402458	MW402813	MW402124	MW402322
F. acutatum	CBS 402.97T	MW402459	MW402768	MW402125	MW402323
F. agapanthi	NRRL 54463T	KU900611	KU900625	KU900630	KU900635
F. ananatum	CBS 118516T	MW402376	LT996137	LT996091	MN534089
F. ananatum	CBS 118517	MN534157	MN534229	MN533988	MN534090
F. andiyaz	CBS 119856	MN534174	MN534286	MN533989	MN534081
F. andiyaz	CBS 119857T	MN534175	LT996138	MN193854	LT996113
F. annulatum	CBS 115.97	MW402373	MW402785	MW401973	MW402173
F. annulatum	CBS 258.54T	MT010908	MT010983	MT010994	MT011041
F. anthophilum	CBS 108.92	MW402368	MW402783	MW401965	MW402166
F. anthophilum	CBS 222.76T	MW402451	MW402811	MW402114	MW402312
F. arcuatisporum	NRRL 32997	GQ505536	GQ505802	GQ505624	
F. awaxy	LGMF 1930T	MK766940	MK766941	MG839004	MG839013
F. bactridioides	CBS 100057T	MN534173	MN534235	MN533993	MN534112
F. bactridioides	NRRL 20476	AF158343		AF160290	U34434
F. begoniae	CBS 403.97	MW402460	MN193886	MN193858	U61543
F. begoniae	CBS 452.97T	MN534163	MN534243	MN533994	MN534101
F. brevicatenulatum	CBS 404.97T		MN534295	MN533995	MN534063
F. brevicatenulatum	CBS 100196		MN193887	MN193859	
F. brevicaudatum	NRRL 43638T	GQ505576	GQ505843	GQ505665	
F. brevicaudatum	NRRL 43694	GQ505579	GQ505846	GQ505668	
F. bubalinum	CBS 161.25T	MN170314	MN170381	MN170448	
F. buharicum	CBS 796.70		JX171563		
F. bulbicola	CBS 220.76T	MW402450	MW402767	KF466415	KF466437
F. caatingaense	CBS 976.97	MN170315	MN170382	MN170449	
F. caatingaense	CBS 130317	GQ505539	GQ505805	GQ505627	
F. callistephi	CBS 187.53T		MH484875	MH484966	MH485057
F. callistephi	CBS 115423		MH484905	MH484996	MH485087
F. carminascens	CBS 144741		MH484936	MH485027	MH485118
F. carminascens	CBS 144738T		MH484937	MH485028	MH485119
F. cateniforme	CBS 150.25T	MN170317	MN170384	MN170451	
F. chinhoyiense	NRRL 25221T	MN534196	MN534262	MN534050	MN534082
F. chinhoyiense	NY 001B5	MN534197	MN534263	MN534051	MN534083
F. circinatum	CBS 405.97T	MN534199	MN534252	MN533997	MN534097
F. circinatum	CBS 119864	MW402389	MW402736	MW401996	MW402196
F. citri	CBS 621.87	MN170318	MN170385	MN170452	
F. citri	CBS 678.77	MN170319	MN170386	MN170453	

F. citri	CBS 130905	MN170320	MN170387	MN170454	
F. clavum	CBS 394.93	GQ505509	GQ505775	GQ505597	
F. clavum	CBS 126202T	MN170322	MN170389	MN170456	
F. clavum	CBS 130395	GQ505547	GQ505813	GQ505635	
F. coffeatum	CBS 635.76T	MN120696	MN120736	MN120755	
F. coffeatum	CBS 430.81	MN120697	MN120737	MN120756	
F. coicis	NRRL 66233T	LT996178	KP083274	KP083251	LT996115
F. compactum	CBS 185.31	GQ505558	GQ505824	GQ505646	
F. compactum	CBS 186.31ET	GQ505560	GQ505826	GQ505648	
F. compactum	NRRL 28029	GQ505514	GQ505780	GQ505602	
F. compactum	NRRL 36318	GQ505558	GQ505824	GQ505646	
F. concentricum	CBS 450.97T	MW402467	JF741086	AF160282	MW402334
F. concentricum	CBS 453.97	MN534216	MN534264	MN533998	MN534123
F. concolor	CBS 961.87T	GQ505585	GQ505852	GQ505645	
F. contaminatum	CBS 111552		MH484900	MH484991	MH485082
F. contaminatum	CBS 114899T		MH484901	MH484992	MH485083
F. croceum	CBS 131777T	MN170329	MN170396	MN170463	
F. croceum	CBS 131788	MN170330	MN170397	MN170464	
F. cugenangense	CBS 130304		MH484921	MH485012	MH485103
F. curvatum	CBS 247.61		MH484876	MH484967	MH485058
F. curvatum	CBS 238.94T		MH484893	MH484984	MH485075
F. denticulatum	CBS 406.97	MN534185	MN534273	MN533999	MN534067
F. denticulatum	CBS 407.97T	MN534186	MN534274	MN534000	MN534068
F. dlaminii	CBS 175.88	MN534150	MN534256	MN534002	MN534138
F. dlaminii	CBS 119860T	MW402388	KU171701	MW401995	MW402195
F. duofalcatisporum	CBS 264.50	GQ505563	GQ505829	GQ505651	
F. duofalcatisporum	CBS 384.92T	GQ505564	GQ505830	GQ505652	
F. duoseptatum	CBS 102026		MH484896	MH484987	MH485078
F. elaeidis	CBS 217.49		MH484870	MH484961	MH485052
F. elaeidis	CBS 218.49		MH484871	MH484962	MH485053
F. equiseti	CBS 107.07	GQ505556	GQ505822	GQ505644	
F. equiseti	CBS 307.94ET	GQ505511	GQ505777	GQ505599	
F. fabacearum	CBS 144742		MH484938	MH485029	MH485120
F. fabacearum	CBS 144743		MH484939	MH485030	MH485121
F. fasciculatum	CBS 131382T	MN170339	MN170406	MN170473	
F. fasciculatum	CBS 131383	MN170340	MN170407	MN170474	
F. ficicrescens	CBS 125177	MN534176	MN534281	MN534006	MN534071
F. ficicrescens	CBS 125178T	KU603958	KT154002	KU604452	KP662896
F. flagelliforme	CBS 162.57 T	GQ505557	GQ505823	GQ505645	
F. flagelliforme	CBS 259.54	GQ505562	GQ505828	GQ505650	
F. foetens	CBS 120665		MH484918	MH485009	MH485100
F. fractiflexum	NRRL 28852T	AF158341	LT575064	AF160288	AF160315
F. fredkrugeri	CBS 144209T	LT996181	LT996147	LT996097	LT996118
F. fujikuroi	CBS 186.56	MW402447	MW402765	MW402108	MW402306

F. fujikuroi	CBS 221.76T		KU604255	MN534010	MN534130
F. globosum	CBS 428.97T	MN534218	KF466406	KF466417	MN534124
F. globosum	CBS 431.97	MW402465	MW402816	MW402131	MW402330
F. glycines	CBS 176.33		MH484868	MH484959	MH485050
F. glycines	CBS 144746		MH484942	MH485033	MH485124
F. gossypinum	CBS 116612		MH484908	MH484999	MH485090
F. gossypinum	CBS 116613T		MH484909	MH485000	MH485091
F. gracilipes	NRRL 43635T	GQ505573	GQ505840	GQ505662	
F. guilinense	NRRL 13335	GQ505502	GQ505768	GQ505590	
F. guilinense	NRRL 32865	GQ505526	GQ505792	GQ505614	
F. guttiforme	CBS 409.97T	MT010901	MT010967	MT010999	MT011048
F. hainanense	CBS 131386	MN170376	MN170443	MN170510	
F. hainanense	CBS 544.96	GQ505510	GQ505776	GQ505598	
F. hoodiae	CBS 132474T		MH484929	MH485020	MH485111
F. hoodiae	CBS 132476		MH484930	MH485021	MH485112
F. humuli	LC 4490	MK289664	MK289767	MK289614	
F. humuli	LC 12158	MK289645	MK289745	MK289592	
F. incarnatum	CBS 132.73NT	MN170342	MN170409	MN170476	
F. incarnatum	CBS 132907	MN170343	MN170410	MN170477	
F. ipomoeae	CBS 135762	MN170344	MN170411	MN170478	
F. ipomoeae	CBS 140909	MN170345	MN170412	MN170479	
F. ipomoeae	LC12162	MK289655	MK289749	MK289596	
F. ipomoeae	LC12164	MK289701	MK289751	MK289598	
F. ipomoeae	LC12165	MK289704	MK289752	MK289599	
F. ipomoeae	LC6926	MK289670	MK289773	MK289619	
F. ipomoeae	NRRL 34034	GQ505548	GQ505814	GQ505636	
F. ipomoeae	NRRL 34039	GQ505551	GQ505817	GQ505639	
F. irregulare	CBS 132190	MN170346	MN170413	MN170480	
F. irregulare	NRRL 31160	GQ505519	GQ505785	GQ505607	
F. irregulare	NRRL 32175	GQ505521	GQ505787	GQ505609	
F. konzum	CBS 119849T	LT996182	MW402733	LT996098	MN534095
F. konzum	CBS 139382	MW402418	MW402804	MW402071	MW402270
F. lacertarum	CBS 130185T	GQ505505	GQ505771	GQ505593	
F. lacertarum	CBS 102300	GQ505555	GQ505821	GQ505643	
F. lactis	CBS 411.97ET	MN534178	MN534275	MN193862	MN534077
F. lactis	CBS 420.97	MN534181	MN534296	MN534015	MN534078
F. languescens	CBS 645.78T		MH484880	MH484971	MH485062
F. languescens	CBS 646.78		MH484881	MH484972	MH485063
F. lateritium Clade I	L-69		AY707155	AY707137	
F. lateritium Clade I	L-83		AY707158	AY707140	
F. lateritium Clade I	L-86		AY707160	AY707142	
F. lateritium Clade I	L-101		AY707163	AY707145	
F. lateritium Clade I	L-375		AY707169	AY707151	
F. lateritium Clade IIA	L-81		AY707156	AY707138	

F. lateritium Clade IIA	L-405		AY707172	AY707154	
F. lateritium Clade IIA	L-200		AY707168	AY707150	
F. lateritium Clade IIB	L-82		AY707157	AY707139	
F. lateritium Clade IIB	L-120		AY707167	AY707149	
F. lateritium Clade III	NRRL 13622	JX171571			
F. lateritium Clade III	L-110		AY707165	AY707147	
F. lateritium Clade III	L-112		AY707166	AY707148	
F. libertatis	CBS 144747		MH484933	MH485024	MH485115
F. libertatis	CBS 144749		MH484944	MH485035	MH485126
F. longicaudatum	CBS 123.73T	MN170347	MN170414	MN170481	
F. longicornicola	NRRL 52706T	MW402487	JF741114	JF740788	MW402360
F. longicornicola	NRRL 52712	MW402488	JF741120	JF740794	MW402361
F. longifundum	CBS 235.79T	GQ505561	GQ505827	GQ505649	
F. luffae	CBS 131097	MN170348	MN170415	MN170482	
F. luffae	NRRL 31167	GQ505520	GQ505786	GQ505608	
F. luffae	LC12167	MK289698	MK289754	MK289601	
F. luffae	NRRL 32522	GQ505524	GQ505790	GQ505612	
F. lumajangense	InaCCF872T		LS479850	LS479441	LS479433
F. lumajangense	InaCCF993		LS479851	LS479442	LS479434
F. madaense	CBS 146648	MW402436	MW402761	MW402095	MW402294
F. madaense	CBS 146669T	MW402439	MW402764	MW402098	MW402297
F. mangiferae	CBS 119853	MN534225	MN534270	MN534016	MN534140
F. mangiferae	CBS 120994T	MN534224	MN534271	MN534017	MN534128
F. massalimae	URM 8239		MN939767	MN939763	MN939759
F. massalimae	FCCUFG 05		MN939768	MN939764	MN939760
F. mexicanum	NRRL 47473	GU737389	LR792615	GU737416	GU737308
F. mexicanum	NRRL 53147T		MN724973	GU737282	GU737494
F. monophialidicum	NRRL 54973	MN170349	MN170416	MN170483	
F. mucidum	CBS 102394	MN170350	MN170417	MN170484	
F. mucidum	CBS 102395T	MN170351	MN170418	MN170485	
F. multiceps	CBS 130386T	GQ505577	GQ505844	GQ505666	
F. mundagurra	RGB5717T	MN534214	KP083276	KP083256	MN534146
F. musae	CBS 624.87T	MW402474	MW402772	FN552086	FN545368
F. musae	NRRL 28893	FN552070	FN552114	FN552092	FN545374
F. nanum	CBS 119867	MN170352	MN170419	MN170486	
F. nanum	CBS 131781	MN170353	MN170420	MN170487	
F. napiforme	CBS 748.97T	MN534192	MN534291	MN193863	MN534085
F. napiforme	CBS 135139	MN534183	MN534290	MN534019	MN534084
F. neoscirpi	CBS 610.95T	GQ505513	GQ505779	GQ505601	
F. neosemitectum	CBS 189.60T	MN170355	MN170422	MN170489	
F. neosemitectum	CBS 190.60	MN170356	MN170423	MN170490	
F. nirenbergiae	CBS 744.97	AF158365	LT575065	AF160312	U34424
F. nirenbergiae	CBS 840.88T		MH484887	MH484978	MH485069
F. nirenbergiae	CBS 115416		MH484902	MH484993	MH485084

F. nygamai	CBS 572.94	MW402473	MW402819	MW402141	MW402341
F. nygamai	CBS 749.97T	MW402479	EF470114	MW402151	MW402352
F. odoratissimum	CBS 102030		MH484898	MH484989	MH485080
F. ophioides	CBS 118509	MN534201	MN534301	MN534020	MN534121
F. ophioides	CBS 118512T	MN534209	MN534303	MN534022	MN534118
F. oxysporum	CBS 144134ET		MH484953	MH485044	MH485135
F. oxysporum	CBS 144135		MH484954	MH485045	MH485136
F. parvisorum	CMW 25267T			KJ541060	KJ541055
F. pernambucanum	CBS 791.70	MN170357	MN170424	MN170491	
F. pernambucanum	CBS 132194	MN170358	MN170425	MN170492	
F. pernambucanum	CBS 132894	MN170359	MN170426	MN170493	
F. persicinum	CBS 479.83T	MN170361	MN170428	MN170495	
F. persicinum	CBS 131780	MN170362	MN170429	MN170496	
F. pharetrum	CBS 144750		MH484951	MH485042	MH485133
F. pharetrum	CBS 144751T		MH484952	MH485043	MH485134
F. phyllophilum	CBS 216.76T	KF466333	KF466410	MN193864	KF466443
F. phyllophilum	CBS 246.61	MW402453		MW402118	MW402316
F. pilosicola	NRRL 29123	MN534165	MN534247	MN534054	MN534098
F. pilosicola	NRRL 29124T	MN534159	MN534248	MN534055	MN534099
F. proliferatum	CBS 480.96T	MN534217	MN534272	MN534059	MN534129
F. pseudoanthophilum	CBS 414.97T	MW402463		MW402128	MW402326
F. pseudoanthophilum	CBS 745.97	MW402476	MW402820	MW402148	MW402349
F. pseudocircinatum	CBS 449.97T	MN534190	MN534277	AF160271	MN534069
F. pseudocircinatum	CBS 455.97	MN534184	MN534276	MN534029	MN534070
F. pseudonygamai	CBS 416.97	MN534194	MN534283	MN534030	MN534064
F. pseudonygamai	CBS 417.97T	AF158316	MN534285	AF160263	MN534066
F. ramigenum	CBS 418.97T	MN534187	KF466412	KF466423	MN534145
F. ramigenum	CBS 526.97	MN534188	MN534292	MN534032	MN534086
F. sacchari	CBS 223.76ET	AF158331	JX171580	MW402115	MW402313
F. sarcochroum	NRRL 20472		JX171586	MW834278	
F. sarcochroum	CPC 26369		LT746320	LT746207	
F. sarcochroum	CPC 27921		LT746323	LT746210	
F. scirpi	CBS 447.84NT	GQ505566	GQ505832	GQ505654	
F. scirpi	CBS 448.84	MN170364	MN170431	MN170498	
F. serpentinum	CBS 119880	MN170365	MN170432	MN170499	
F. siculi	CBS 142222T	LT746189	LT746327	LT746214	LT746346
F. sterilihyposum	NRRL 25623T	AF158353	MN193897	MN193869	AF160316
F. sterilihyposum	NRRL 53991	GU737386		GU737413	GU737305
F. stilboides	NRRL 20429		JX171582		
F. subglutinans	CBS 215.76	MN534171	MN534241	MN534061	MN534109
F. subglutinans	CBS 747.97NT	MW402478	MW402773	MW402150	MW402351
F. succisae	CBS 187.34	MW402448	MW402810	MW402109	MW402307
F. succisae	CBS 219.76ET	AF158344	MW402766	AF160291	U34419
F. sudanense	CBS 454.97T	MN534179	MN534278	MN534037	MN534073

F. sudanense	CBS 675.94	MN534182	MN534279	MN534038	MN534074
F. sulawesiense	CBS 131.73	MN170366	MN170433	MN170500	
F. sulawesiense	CBS 122439	MN170370	MN170437	MN170504	
F. sulawesiense	InaCC F940T	LS479422	LS479855	LS479443	
F. tanahbumbuense	CBS 145.44	MN170371	MN170438	MN170505	
F. tanahbumbuense	CBS 131009	MN170372	MN170439	MN170506	
F. tanahbumbuense	InaCC F965T	LS479432	LS479863	LS479448	
F. temperatum	MUCL 52463T	MW402486	MW402776		MW402359
F. terricola	CBS 483.94T	MN534189	LT996156	MN534042	MN534076
F. terricola	CBS 119850	MN534180	MN534280	MN534041	MN534075
F. thapsinum	CBS 539.79	MW402472	MW402818	MW402140	MW402340
F. thapsinum	CBS 776.96T		MN534289	MN534044	MN534080
F. tjaetaba	NRRL 66243T	LT996187	KP083275	KP083263	
F. toxicum	CBS 219.63	MN170373	MN170440	MN170507	
F. toxicum	CBS 406.86T	MN170374	MN170441	MN170508	
F. trachichlamydosporum	CBS 102028		MH484897	MH484988	MH485079
F. triseptatum	CBS 258.50T		MH484873	MH484964	MH485055
F. triseptatum	CBS 116619		MH484910	MH485001	MH485092
F. tupiense	NRRL 53984T	GU737377	LR792619	GU737404	GU737296
F. udum	CBS 178.32	MW402442	LT996172	AF160275	U34433
F. udum	CBS 177.31		MH484866	MH484957	MH485048
F. udum	NRRL 25199ET		KY498875	KY498862	KY498892
F. verticillioides	CBS 125.73	MW402392	MW402791	MW402012	MW402212
F. verticillioides	CBS 218.76ET	MW402449		MW402113	MW402311
F. veterinarium	CBS 109898T		MH484899	MH484990	MH485081
F. veterinarium	CBS 117787		MH484912	MH485003	MH485094
F. volatile	CBS 143874T	MK984595	LR596006	LR596007	LR596008
F. volatile	NRRL 25615	AF158357		AF160304	AF160320
F. werrikimbe	CBS 125535T	MN534203	MN534304		MN534104
F. xylarioides	CBS 258.52T	MW402455	HM068355	MN193874	AY707118
F. xyrophilum	NRRL 62721T		MN193905	MN193877	
